# Supplementary material for: Surfing beta burst waveforms to improve motor imagery-based BCI
Source: Imaging Neurosci (Camb). 2024 Dec 16;2:imag-2-00391. doi: 10.1162/imag_a_00391 (PMC12315766; doi:10.1162/imag_a_00391)
Supplement: Supplementary Material [file imag_a_00391-supp.pdf]

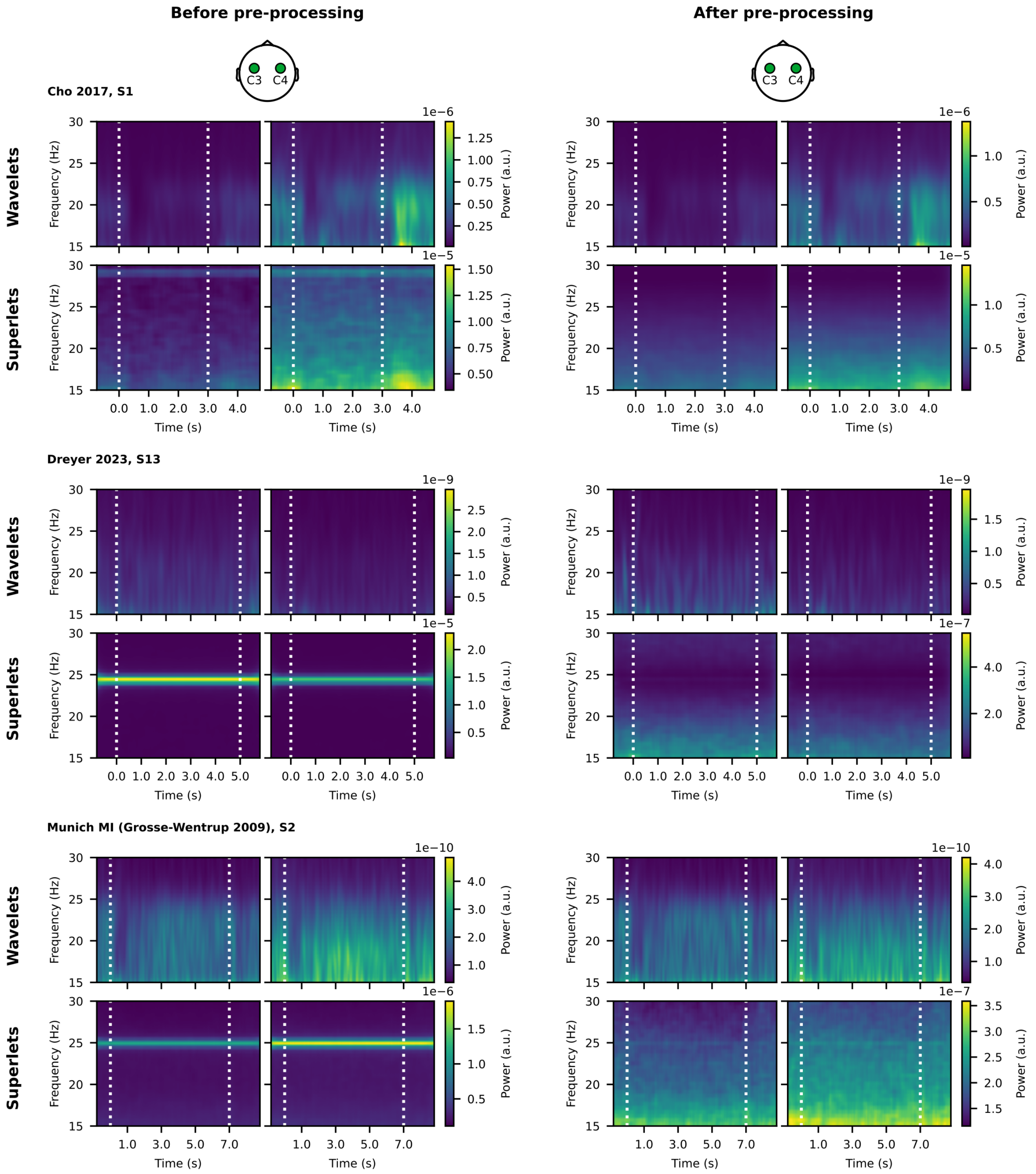

Sup. Figure 1: Three examples of trial-averaged data contaminated by noise in the frequency range of 24 – 30 Hz. The first column shows contaminated data in channels C3 and C4. The narrow-band, high-amplitude oscillation is present in both hemispheres and is not modulated by task demands (task onset and end are indicated by the dotted, vertical lines). In every example, a wavelet-based, time-frequency transformation does not reveal the underlying noise, unlike the superlets transformation which offers better time and frequency resolution. The second column showcases that the pre-processing pipeline effectively cleans the recordings allowing for detection of the underlying burst activity.

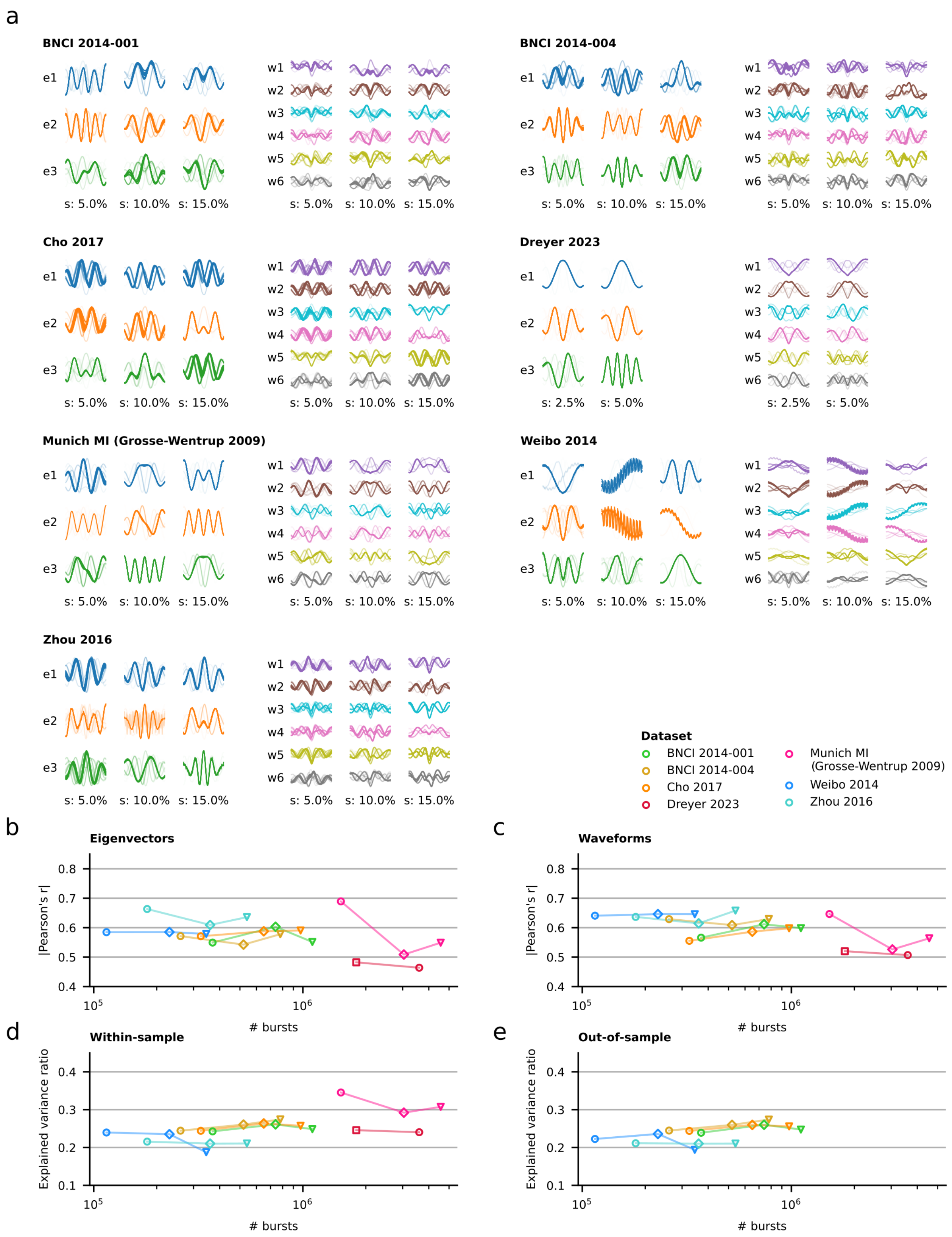

Sup. Figure 2: Collection of PCA eigenvectors and corresponding waveforms used as kernels, selected using the modulation index. **(a)** For each dataset each column shows the eigenvectors (e1-3) and waveforms (w1-6) corresponding to a different sample size (s). The random sampling per sample size is repeated thirty times. Within each subplot, Pearson's correlation coefficients are used to rank the selected eigenvectors and waveforms per iteration of the random resampling. The shape with the maximum absolute correlation to the corresponding shape of the first iteration is plotted. Transparency levels of the depicted shapes equal the corresponding absolute correlation

coefficients. The polarity of the depicted shapes is determined based on the signed correlation coefficients and matches the polarity of the first iteration's shapes. Smaller sample sizes are often characterized by greater variability in the shape of the selected eigenvectors and waveforms. The shapes of the selected eigenvectors and waveforms are more consistent when the sample size increases. High-frequency noise remaining in the data after pre-processing may affect some of the selected eigenvectors and waveforms, most prominently in the case of the Weibo 2014 dataset. **(b)** Sum of absolute Pearson's correlation coefficients across all selected eigenvectors, averaged over all random samples. Different sample sizes are quantified in terms of the number of bursts used for fitting the PCA algorithm. Higher values imply higher reproducibility of selected eigenvectors. A value of 1 corresponds to identical eigenvectors and a value of 0 to orthogonal eigenvectors. Within each dataset values remain stable regardless of the increase in the number of bursts included in the PCA, especially for datasets with fewer number of detected bursts. **(c)** Average of maximum absolute Pearson's correlation coefficients across all selected waveforms, averaged over all random samples. Higher values imply higher reproducibility of selected waveforms. The sample size used in this study offers consistent results in kernel selection. **(d)** Variance ratio of randomly sampled bursts explained by the selected eigenvectors, averaged over all random samples. **(e)** Variance ratio of all detected bursts explained by the selected eigenvectors, averaged over all random samples. The amount of data for two of the datasets included in this study was too large to perform this evaluation. The selected eigenvectors explain approximately the same amount of data variability irrespective of the tested sample size. Markers indicate the sample size as percentage of the total available trials (□ : 2.5%, o : 5%, ♦ : 10%, ᵛ : 15%). The values on the abscissa are plotted on a logarithmic scale. Each dataset is color-coded with a distinct color as indicated in the legend.

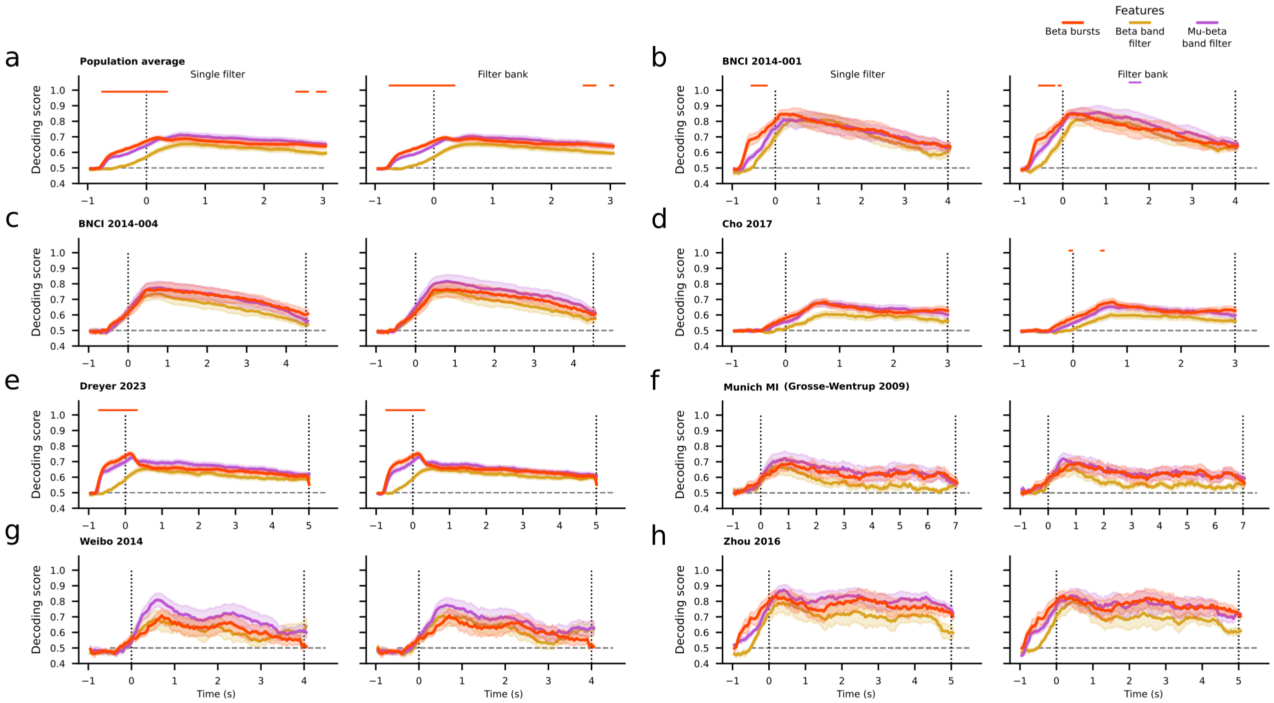

Sup. Figure 3: (a) Population average, time-resolved decoding score and standard error for the beta burst convolution (red), beta band (yellow) and mu-beta band (purple) filtering pipelines using a sliding window. Due to the different duration of the task for each dataset we restricted the time to the minimum trial period corresponding to 3 seconds. **(b – h)** Average, time-resolved decoding score and standard error per dataset of the same features using a sliding window. For each panel, the left subplot depicts the decoding results obtained using a single filter, while the right subplot depicts the results based on a filter bank technique. The beta burst results are the same for the pair of each panel. The horizontal dashed line corresponds to the expected chance level. Vertical dotted lines represent the onset and end of the trial period of each dataset. The two horizontal lines on the top of each subplot show the results of the two pair-wise permutation cluster tests, that is, between the beta bursts and the beta band (bottom line) or mu-beta band (top line) filtering technique respectively, with correction for multiple comparisons at significance level of 0.05. At any time point, each line is color-coded so as to indicate which feature produces, on average, better results. A lack of color at any given time point indicates no statistically significant differences between the compared features.

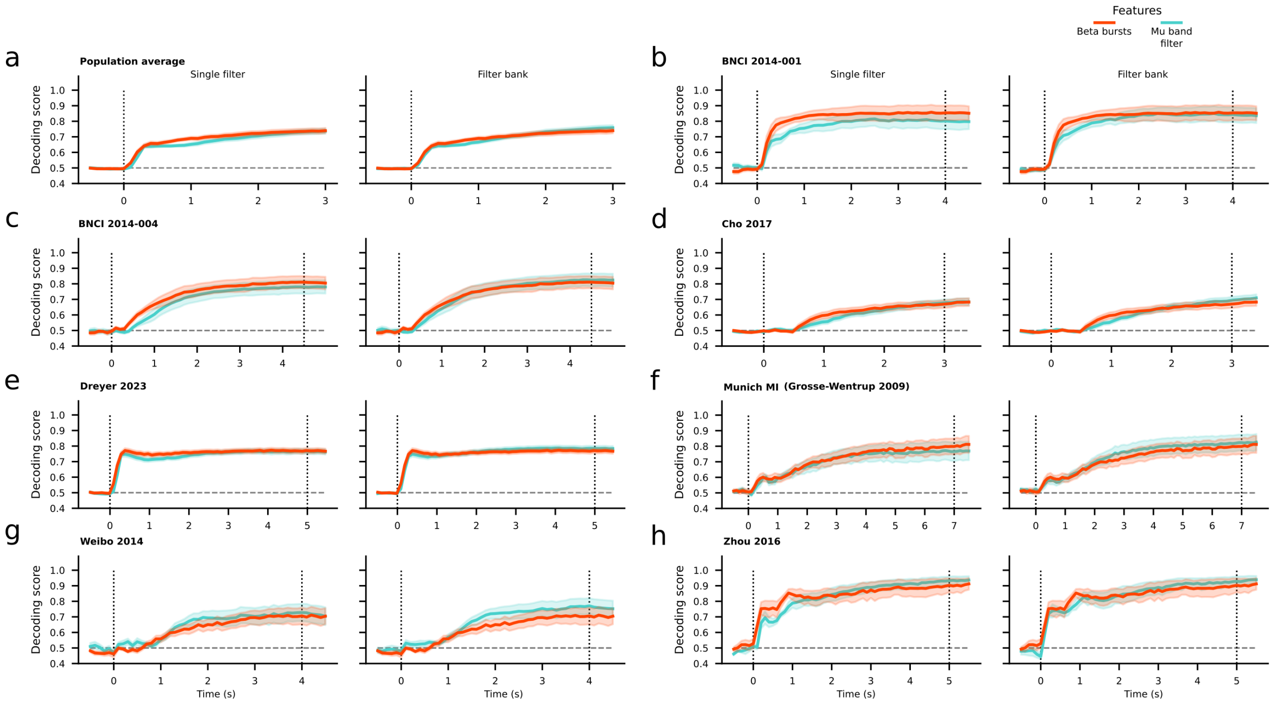

Sup. Figure 4: (a) Population average, time-resolved decoding score and standard error for the beta burst convolution (red) and mu band (turquoise) filtering pipelines using an incremental window. Due to the different duration of the task for each dataset we restricted the time to the minimum trial period corresponding to 3 seconds. **(b – h)** Average, time-resolved decoding score and standard error per dataset of the same features using an incremental window. For each panel, the left subplot depicts the decoding results obtained using a single filter, while the right subplot depicts the results based on a filter bank technique. The beta burst results are the same for the pair of each panel. The horizontal dashed line corresponds to the expected chance level. Vertical dotted lines represent the onset and end of the trial period of each dataset. The horizontal line on the top of each subplot shows the results of the pair-wise permutation cluster tests that is, between the beta bursts and the mu band filtering technique, with correction for multiple comparisons at significance level of 0.05. At any time point, each line is color-coded so as to indicate which feature produces, on average, better results. A lack of color at any given time point indicates no statistically significant differences between the compared features.

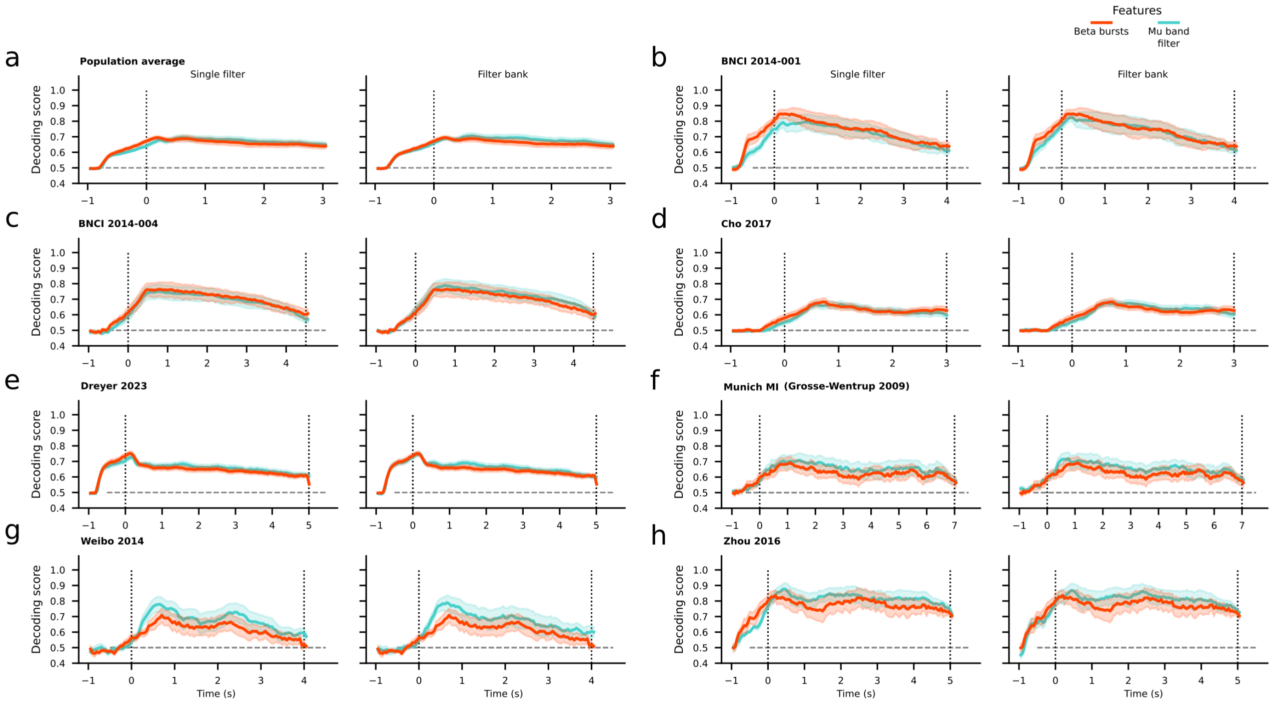

Sup. Figure 5: (a) Population average time-resolved decoding score and standard error for the beta burst convolution (red) and mu band (turquoise) filtering pipelines using a sliding window. Due to the different duration of the task for each dataset we restricted the time to the minimum trial period corresponding to 3 seconds. **(b – h)** Average, time-resolved decoding score and standard error per dataset of the same features using a sliding window. For each panel, the left subplot depicts the decoding results obtained using a single filter, while the right subplot depicts the results based on a filter bank technique. The beta burst results are the same for the pair of each panel. The horizontal dashed line corresponds to the expected chance level. Vertical dotted lines represent the onset and end of the trial period of each dataset. The horizontal line on the top of each subplot shows the results of the pair-wise permutation cluster tests that is, between the beta bursts and the mu band filtering technique, with correction for multiple comparisons at significance level of 0.05. At any time point, each line is color-coded so as to indicate which feature produces, on average, better results. A lack of color at any given time point indicates no statistically significant differences between the compared features.

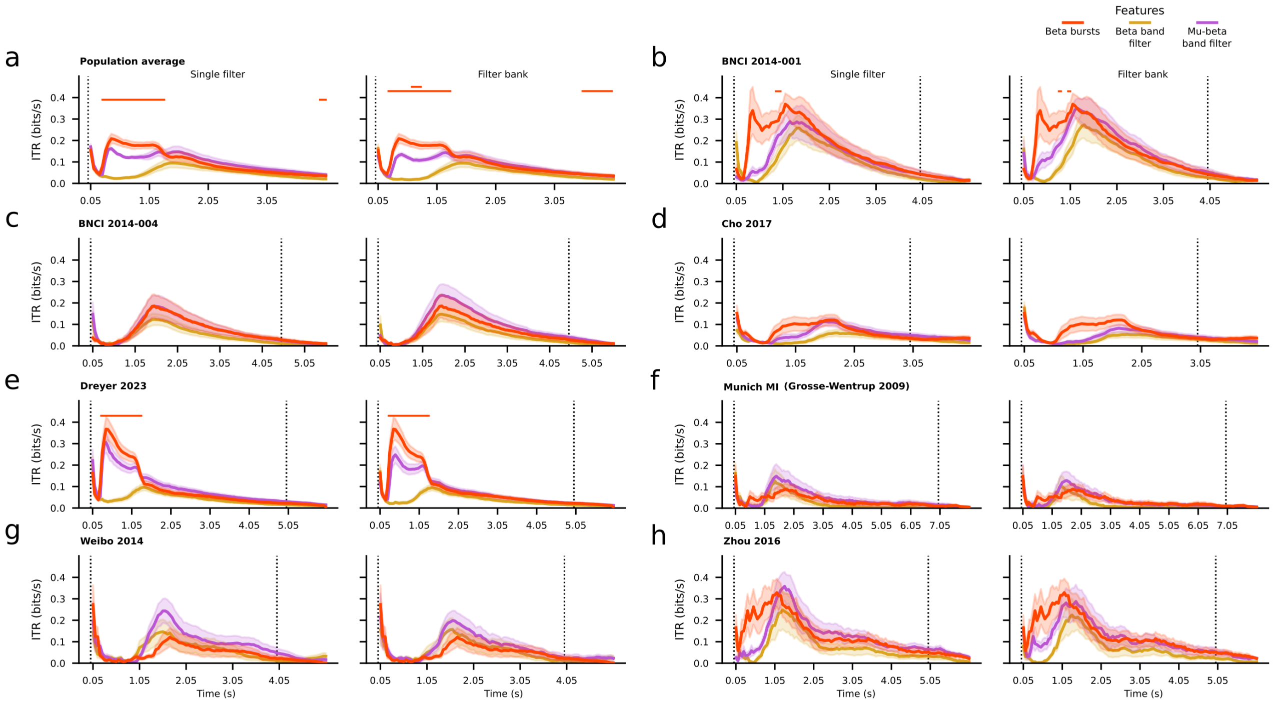

Sup. Figure 6: (a) Population average, time-resolved information transfer rate (ITR) and standard error for the beta burst convolution (red), beta band (yellow) and mu-beta band (purple) filtering pipelines using a sliding window. Due to the different duration of the task for each dataset we restricted the time to the minimum trial period corresponding to 3 seconds. **(b – h)** Average, time-resolved information transfer rate (ITR) and standard error per dataset of the same features using a sliding window. For each panel, the left subplot depicts the ITR results obtained using a single filter, while the right subplot depicts the results based on a filter bank technique. The beta burst results are the same for the pair of each panel. Vertical dotted lines represent the onset and end of the trial period of each dataset. The two horizontal lines on the top of each subplot show the results of the two pair-wise permutation cluster tests, that is, between the beta bursts and the beta band (bottom line) or mu-beta band (top line) filtering technique respectively, with correction for multiple comparisons at significance level of 0.05. At any time point, each line is color-coded so as to indicate which feature produces, on average, better results. A lack of color at any given time point indicates no statistically significant differences between the compared features.

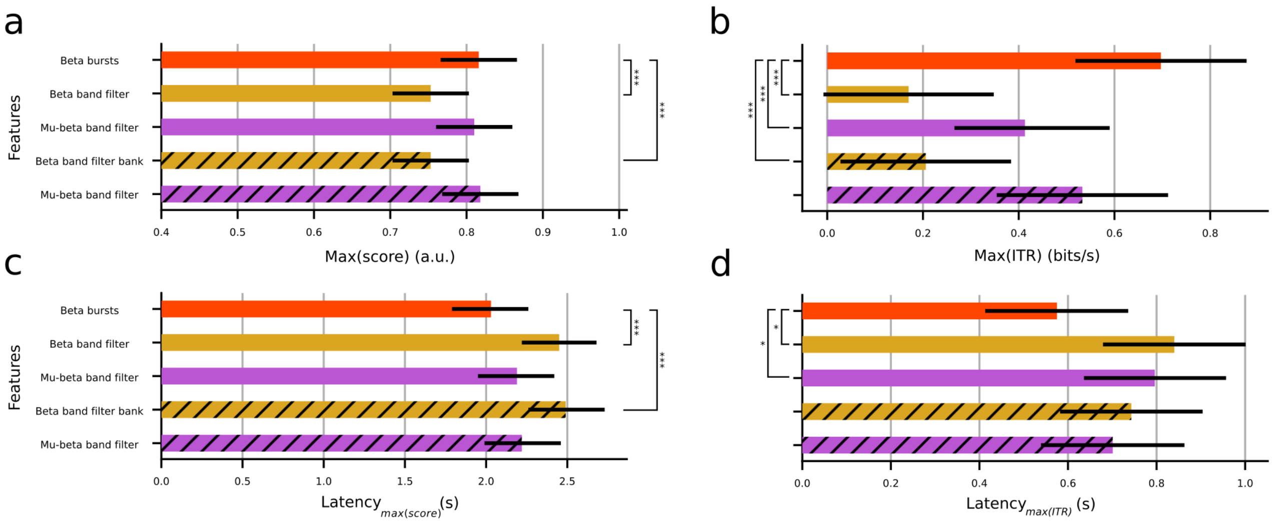

Sup. Figure 7: Population-level statistical analysis based on linear mixed models when using a sliding window per feature. **(a)** Average maximum decoding score. **(b)** Average maximum ITR. **(c)** Average latency to reach the maximum decoding score. **(d)** Average latency to reach the maximum ITR. Error bars show 95% confidence intervals. Hatches indicate the use of a filter bank technique. Asterisks indicate statistically significant differences among pairwise comparisons of the beta bursts and the rest of the features (* : $p < 0.05$. ** : $p < 0.01$, *** : $p < 0.001$). A lack of asterisks implies no statistically significant differences. Note that the log transform of the the maximum ITR and latency to maximum ITR were used for the statistical analysis (see Methods), but panels **b** and **d** depict results before applying the transformation for ease of comparisons with panels **a** and **c** respectively.
